# Tuberculosis of the Cervical Spine: A Case Report

**DOI:** 10.7759/cureus.44911

**Published:** 2023-09-08

**Authors:** Dhriti Jain, Ventaktesh Dasari, Nikhil Kaushik, Garima Singh

**Affiliations:** 1 Medicine, Jawaharlal Nehru Medical College, Datta Meghe Institute of Medical Sciences, Wardha, IND; 2 Orthopedics and Traumatology, Jawaharlal Nehru Medical College, Datta Meghe Institute of Medical Sciences, Wardha, IND

**Keywords:** corpectomy, management, spine, cervical, tuberculosis

## Abstract

Tuberculosis of the cervical spine is a rare but deadly form of tuberculosis (TB), where the infection affects the cervical vertebrae or bones of the neck. It is also known as Pott’s disease. The causative organism is Mycobacterium tuberculosis. It usually occurs when TB bacteria spread from other body parts, such as the lungs, to the cervical vertebrae through the bloodstream or lymphatic system. It also shows extrapulmonary involvement, including the central nervous, genitourinary, and lymphatic systems, bones, and joints. Tuberculosis of the spine is often seen. Cervical spine tuberculosis is a rare form of the disease though. If the infection is not treated, it might harm the spinal cord and nerves, resulting in paralysis and other neurological complications. This article presents a 40-year-old female with tuberculosis of the cervical spine complaining of neck pain and stiffness with neurological manifestations and its management with anterior spinal instrumentation. The patient showed improvement in the next follow-up.

## Introduction

Tuberculosis is considered a serious and significant health condition in the world. The burden of tuberculosis in 2021 was approximately 10.6 million people worldwide. Among all the cases of TB, musculoskeletal manifestation like spinal tuberculosis is said to comprise 1% to 2% [1]. Tuberculosis of the cervical spine is again a rare condition with an occurrence rate of 2% to 5% among spinal cases [2]. Patients with cervical spine tuberculosis usually present with neck pain, neck stiffness, fever, night sweats, weight loss, and neurological symptoms such as weakness or numbness in the arms or legs. The treatment modalities for cervical spine tuberculosis include medical and surgical management. We report a case of tuberculosis of the cervical spine with neurological symptoms managed surgically.

## Case presentation

A 40-year-old female, homemaker by occupation, presented with the chief complaint of neck pain and stiffness for one month. She had a history of pulmonary tuberculosis, which was treated with a six-month course of anti-tuberculosis medications one year ago. The Mycobacterium culture was positive, hence the treatment was taken. It was not a case of multidrug-resistant TB (MDR TB). On asking further questions, she gave an account of weakness in all four limbs. There was a history of weight loss and decreased appetite. There was no bowel or bladder dysfunction. On general examination, she was afebrile, her pulse was 90 beats per minute, her blood pressure was 120/80 mmHg, and her respiratory rate was 20 per minute. On physical examination, the patient was conscious, cooperative, and well-oriented to time, place, and person. There was a paraspinal spasm. On neurological examination, the following results were obtained. The power of both the upper limbs was 3/5. The control of both the lower limbs was 4/5. Plantar reflex was positive on both sides. Hyperactive deep tendon reflexes were seen in the upper and lower limbs. There was no sensory loss. The modified Japanese Orthopaedic Association (mJOA) score was 16. On local examination, there was spinal tenderness at the C5-C7 level. Paraspinal muscle tenderness was observed. The American Spinal Injury Association (ASIA) score was E. On laboratory tests, there was an increase in white blood cells (WBC) and C-reactive protein levels (15-10*3/mm^3 and 7.9 mg/dl, respectively).

Diagnostic assessment

**Figure 1:**
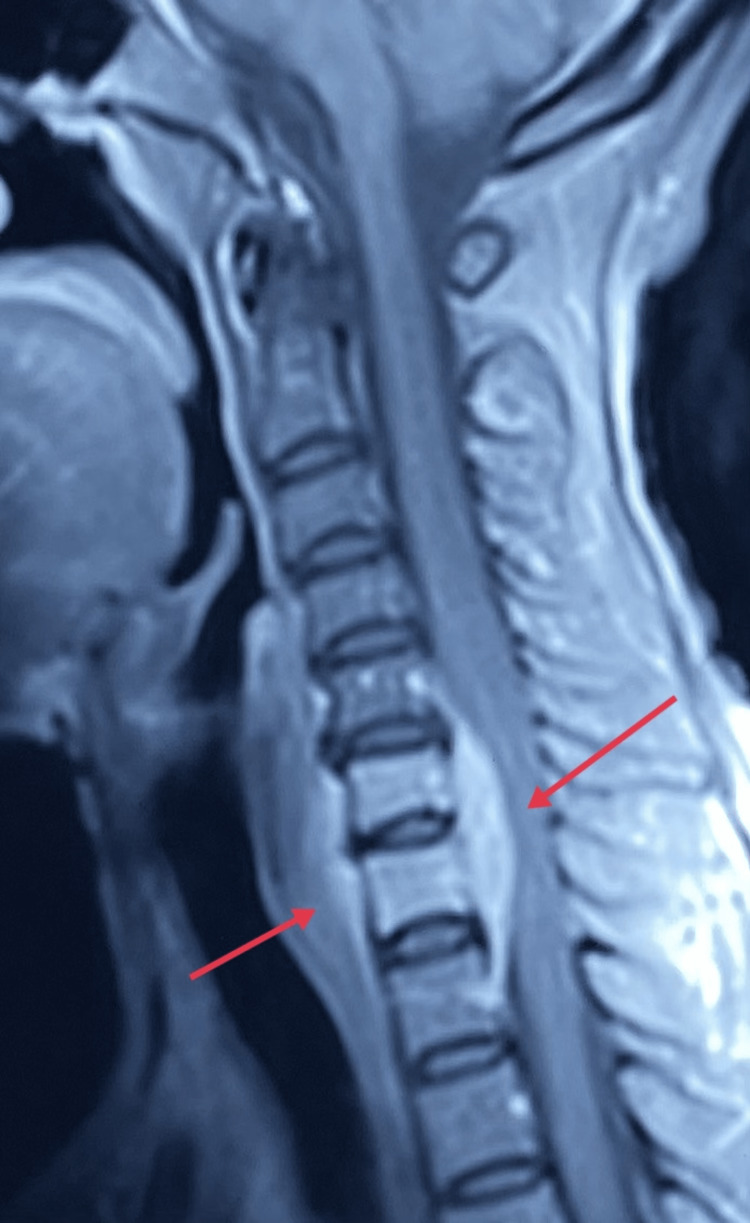
Red arrows showing subtle cord edema is seen at the C6-C7 level

Magnetic resonance imaging (MRI) plain and contrast of the cervical spine showed mild straightening of the cervical spine. Abnormal signals are seen in the C6 and C7 vertebral bodies, and the C6-C7 disc appears hypointense on T1 W images and hyper-intense on T2 W/STIR images with abnormal post-contrast enhancement. Abnormal pre and paravertebral soft tissue is seen from C6 to D1 levels. Abnormal anterior epidural soft tissue is seen from C5 to D1 levels (maximum at C6-C7), measuring approximately 6 mm thickness, compressing the anterior subarachnoid space and ventral surface of the spinal cord. Subtle cord edema is seen at the C6-C7 level as shown in Figure 1. Changes like cervical spondylosis are seen. A posterior bulge is seen at the C5-C6 level, indenting the anterior subarachnoid space and mildly encroaching over the bilateral neural foramina. The rest of the intervertebral discs reveal no significant bulge or herniation. The rest of the spinal cord shows no abnormal signals. The craniovertebral junction appears normal. There is no evidence of atlantoaxial subluxation/ dislocation.

Treatment

**Figure 2:**
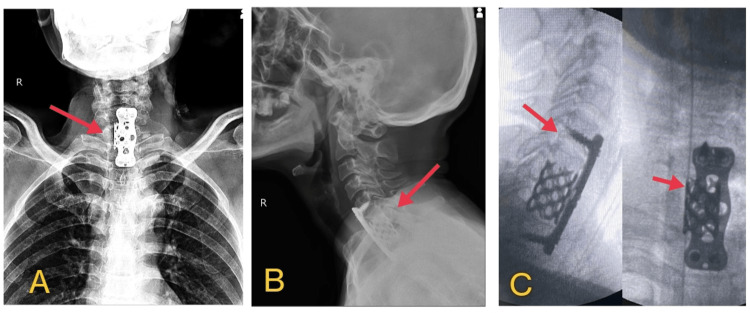
Radiographic images with arrows showing anterior cervical spine instrumentation with plates and screws A. X-ray PA view (posteroanterior view) B. X-ray lateral view C. X-ray showing plates and screws.

Considering the significant motor weakness, upper motor neuron type of quadriparesis, and high mJOA scores, the spine board suggested going ahead with the patient's surgical management. The patient was managed with corpectomy and anterior cervical fusion under general anesthesia. During the procedure, the patient was taken to a spine table. Under general anesthesia, the neck was cleaned and draped and the anterior cervical approach was taken under radiological guidance over the C6-C7 levels after soft tissue dissection. A corpectomy of the C7 vertebral body and a hemi-corpectomy of the inferior part of the C6 vertebral body was performed. During the process of corpectomy, frank pus was obtained from the surgical site, which was collected in a sterile container and sent for further investigations. Thorough anterior decompression of the cord was done. A thorough lavage was given, and the site was prepared for grafting. A dry cortical graft of cancellous bone was obtained from the iliac crest, and the prepared bone graft and a Moss-Miami cage were placed at the prepared corpectomy site. After satisfactory cage placement, an anterior H-plate was placed over the hemicorpectomized C5 and T1 vertebrae. The plate was secured in place using screws as shown in Figure 2.

Further lavage was given, and closure was done in layers. The patient was extubated, and the postop was uneventful. During the postop recovery rehabilitation protocol, the patient was given a Philadelphia collar for the initial two weeks, which was later shifted to a hard cervical collar and gradually to a soft cervical collar over three months, depending on the radiological and clinical progression of the patient. During the postop rehabilitation care, the patient was counseled and re-educated regarding posture correction and advised for general conditioning exercises like walking and specific muscle group strengthening exercises like upper and lower limb strengthening exercises, back strengthening, and extension exercises. The patient was mobilized on postop day 1 itself, as the patient showed good clinical recovery postoperatively. Gradually, over the follow-up of three months, the patient showed significant improvement in neck pain and the neurological components of motor grading and upper motor neuron lesion in due time. The intra-op purulent sample was collected along with the necrotic bone tissue and was sent for further investigations. A direct smear test was done and found to be negative. As part of the protocol, the sample was sent for gene Xpert, and an MTB-RIF test was performed and showed no signs of rifampicin resistance. A quantiferon gold TB test was also performed, which came out positive, and hence the decision was taken to start the patient on Cat 1 AKT as per the DOTS (directly-observed treatment, short-course) protocol to be continued for 18 months, as mentioned in Table 1. As part of the postop medication regime, the patient was managed with IV antibiotics for three days (ceftriaxone 1 g twice daily), analgesia as per the requirement, and supplementation in the form of multivitamins, pyridoxine, high protein diet, and vitamin C. For the management of neurological outcomes, the patient was started on supplementation of methylcobalamin 750 mcg twice daily for three months, pregabalin 75 mg twice daily for the radicular pain, duloxetine 20 mg twice daily for the paresthesia, and baclofen 20 mg twice daily for upper motor neuron related spasticity. All these neurological drugs were gradually tapered off over the course of three months. The patient showed significant neurological improvement with rehabilitation and medication postoperatively. During her hospital stay, her motor grading increased from grade 2 to grade 3/3+, and by her three-month follow-up, the power charting had increased to 4/4+. The detailed power charting has been mentioned below in Table 2.

**Table 1:** Anti-tubercular drug regime as per the DOTS protocol H - isoniazid, R - rifampicin, Z - pyrazinamide, E - ethambutol, S - streptomycin, DOTS - directly-observed treatment, short-course The number before the phase in the bracket indicates the duration of that phase in months. The number after the bracket indicates the number of doses of that drug per week.

Category	Regimen	Duration in months
Category 1	2 (HRZE)3, 4 (HR)3	6
Category 2	2 (HRZES)3, 1 (HRZE)3, 5 (HRE)3	8
Category 3	2 (HRZ)3, 4 (HR)3	6

**Table 2:** Grading of power

Score	Description
1	No contraction
2	Flicker or trace of contraction
3	Active movement against gravity
4	Active movement against gravity and resistance
5	Normal power

## Discussion

Our patient presented with complaints of neck stiffness and weakness in all four limbs, likely secondary to tuberculosis infection. The patient also had weight loss. Further evaluation shows a loss of power in all the limbs. Tuberculosis is one of the deadliest diseases in developing countries [3]. Among all the tuberculosis cases, cervical spine tuberculosis accounts for 3-5% [3,4]. The cervical spine is more susceptible to neurodegeneration, instability, and progressive misalignment because the dimension of the canal is small, and its proximity to the vertebral artery and other critical structures, distinctive facetal architecture, increased mobility, and lordotic alignment [3-6]. The patients are usually middle-aged [4]. The most severe manifestation among such patients is neurological deficits [7]. The patients with cervical spine tuberculosis present with the clinical symptoms of neck pain and stiffness [4]. Our patient had neck pain, stiffness, and weakness in all four limbs. The first investigation to be done to diagnose the disease is magnetic resonance imaging because it is more sensitive and specific than a CT scan [2]. The various treatment methods include surgical and conservative approaches. Medical management alone should be considered in the absence of neurological deficits and combined with surgical treatment in case of deficits. Patients with severe complications like extensive bone involvement, neurological symptoms, spinal deformity, and resistance to a conservative approach should be treated with surgery [8]. The best treatment option for this condition was anterior spinal instrumentation following decompression [9]. However, options for percutaneous decompression could have been discussed, as there was no instability yet. In this case, our patient was treated with a corpectomy of C7, hemicorpectomy of inferior C6, and anterior cervical fusion. The patient showed significant improvement within three months of the surgery.

## Conclusions

Tuberculosis is one of the most prevalent diseases. It can have pulmonary as well as extrapulmonary manifestations. Cervical spine tuberculosis is a rare condition that accounts for very few spinal cases. It can present with neurological complications as well. The treatment option for this case was a surgical method, which included cord decompression with corpectomy, anterior spinal instrumentation, and bone grafting. Also, an anti-tubercular regime was started postoperatively.
